# The Effects of Time Lag and Cure Rate on the Global Dynamics of HIV-1 Model

**DOI:** 10.1155/2017/8094947

**Published:** 2017-06-13

**Authors:** Nigar Ali, Gul Zaman, Abdullah, Aisha M. Alqahtani, Ali Saleh Alshomrani

**Affiliations:** ^1^Department of Mathematics, University of Malakand, Chakdara Dir (L), Khyber Pakhtunkhwa, Pakistan; ^2^Department of Mathematics, Faculty of Science, Jiangsu University, Zhenjiang, Jiangsu 212013, China; ^3^Department of Mathematics, Princess Nourah bint Abdulrahman University, Riyadh, Saudi Arabia; ^4^Department of Mathematics, Faculty of Science, King Abdul Aziz University, Jeddah, Saudi Arabia

## Abstract

In this research article, a new mathematical model of delayed differential equations is developed which discusses the interaction among CD4 T cells, human immunodeficiency virus (HIV), and recombinant virus with cure rate. The model has two distributed intracellular delays. These delays denote the time needed for the infection of a cell. The dynamics of the model are completely described by the basic reproduction numbers represented by *R*_0_, *R*_1_, and *R*_2_. It is shown that if *R*_0_ < 1, then the infection-free equilibrium is locally as well as globally stable. Similarly, it is proved that the recombinant absent equilibrium is locally as well as globally asymptotically stable if 1 < *R*_0_ < *R*_1_. Finally, numerical simulations are presented to illustrate our theoretical results. Our obtained results show that intracellular delay and cure rate have a positive role in the reduction of infected cells and the increasing of uninfected cells due to which the infection is reduced.

## 1. Introduction

Human immunodeficiency virus (HIV) is a virus that causes the condition of acquired immunodeficiency syndrome (AIDS). The virus attacks a particular type of immune system cell in the body, known as CD4 helper lymphocyte cells. HIV destroys these cells, making it harder for body to fight off other infections. Without treatment HIV-1 infection passes through three different phases for HIV-1 infection without treatment. The first one is the primary infection, the second is chronic infection, and the third is acquired immunodeficiency syndrome (AIDS). Several scientists and researchers are working globally to investigate an effective way to cure AIDS but they failed to completely eliminate immunodeficiency virus from the human body.

In the recent past, mathematical modeling was often used to study in vivo infection dynamics of many viruses such as HIV-I, HBV, and HCV. Researchers have gained much knowledge from these models about the mechanism of the interactions of different components such as infected cells and immune system within a host and have thereby enhanced the progress in understanding the HIV-1 infection. Such understanding in turn may offer guidance for developing new drugs and for designing optimal combination of existing therapies. The basic and simple model of HIV-1 infection consisting of three populations, uninfected cells, infected cells, and viral particles, is governed by the following three-dimensional model of nonlinear ordinary differential equations (ODEs) [1–3]:

(1) x˙t=λ−dxt−βxtvt,y˙t=βxtvt−ayt,v˙t=kyt−pvt.

The different densities of uninfected cells and infected cells and the density of virus have been denoted by *x*(*t*), *y*(*t*), and *v*(*t*), respectively. *λ* is the rate at which new susceptible cell is generated. *d* is natural death rate of uninfected cells and *β* is the rate of infection. *a* is the death rate of infected cells which produce new virus particles at a rate *k*. Rong et al. [4] extended the basic model of HIV-1 infection to four-dimensional ordinary differential equation model, where latent period for the infected cells is included and a portion of these cells is reverted to the uninfected class. This improvement makes the use of nonlinear stability methods nontrivial. The authors only established the local asymptotic stability of the equilibria. However, they left the global stability of the model as an open problem. Buonomo and Vargas-de-León [5] resolved elegantly the issue left in [4] and obtained the conditions of global stability of the equilibrium states by using two distinct techniques: Lyapunov direct method and Li and Muldowney's geometric approach. In fact, to build up a more beneficial understanding of a virus dynamics in vivo, so many authors have been devoted to studying the mechanism of infected cells reverting to the uninfected state by loss of all cccDNA from their nucleus, (see Tian and Liu [6]). Conclusive evidences that infected CD4 T cells could be cured by chemotherapy can be found in [7, 8], which is one of our motivations in the modeling for viral dynamics.

In different control measures, recombinant virus is one which is used for controlling the infection of HIV-1 [9–13]. The decline of HIV-1 load about 1000-fold has been proved using recombinant in vitro studies. But the efficacy of this control strategy for decreasing the viral load in AIDS patients is unknown. Genetic engineering offers an alternative approach, featuring modification of a viral genome to produce recombinant capable of controlling infections by other viruses [11]. This method has been used to modify rhabdoviruses, including the rabies and the vesicular stomatitis viruses (VSV), making them capable of infecting and killing cells previously attacked by HIV-1. The engineered virus codifies the preceptor pair CD4 and CXCR4 of the host cell membrane and bind to the protein complex gp120/41 of HIV-1 expressed on the surface of infected cells [9]. A basic estimation using a currently engineered virus indicated an HIV-1 load reduction of 9 percent and a recovery of host cells to 17 percent of their normal level. Greater success (98 percent HIV reduction, 44 percent host cells recovery) is expected as more competent engineered viruses are designed. These results suggest that therapy using viruses could be an alternative to extend the survival of AIDS patients. The purpose of introducing this virus is to fight with HIV to control this infection. In [11], a new virus was introduced into model (1) and the model modified to the following form:

(2) x˙t=λ−dxt−βxtvt,y˙t=βxtvt−ayt−αwtyt,z˙t=awtyt−bzt,v˙t=kyt−pvt,w˙t=czt−qwt.

Here the new variables *w*(*t*) and *z*(*t*) stand for recombinant virus and double-infected cells, respectively. The rate of production of double-infected cells is *α*. The removal rate of recombinant is denoted by *qw*. *bz* is the death rate of double-infected cells which release recombinant at a rate *cz*. The authors of the above model analyzed the structure of equilibrium solutions and presented some simulations. Further, Jiang et al. [12] completely analyzed this model. Yu and Zou [13] modified model (2) by incorporating a control parameter *η* to measure the injection rate of the recombinant for controlling/eliminating the HIV virus. Tian et al. [14] modified this model further by introducing the time lag into model (2) because there is time lag in infection process. They extended model (2) by introducing time delay and studied the effect of delay in controlling this infection.

In this paper, we consider that the contact process between the uninfected and virus-producing cells is not instantaneous. Thus, we include a delay, similar to the disease transmission term, in the rate of contact term. Further we also incorporate recovery rate of unproductively infected cells to uninfected cells. The recovery of these cells to uninfected cells is due to loss of all DNA from their nucleus by using drugs therapy [15, 16]. Our proposed model is extended to the following model after incorporating the above-mentioned terms:

(3) x˙t=λ−dxt−βe−aτxt−τvt−τ+γyt,y˙t=βe−aτxt−τvt−τ−a+γyt−αwtyt,z˙t=αwtyt−bzt,v˙t=kyt−pvt,w˙t=czt−qwt,

where *γ* is the rate of reversion of infected cells. *τ* denotes time lag in contact and infection process. We present the dynamical behavior of the proposed model and show how delays and cure rate influence stability. We prove the well-posedness of the proposed model and study the effect of delay and cure rate in controlling HIV-1. We find the basic reproduction numbers. It is shown that infection-free equilibrium *E*_0_ is locally as well as globally asymptotically stable. It is also shown that *E*_1_ (recombinant absent equilibrium) is locally as well as globally asymptotically stable.

We have divided this paper into the following sections. The well-posedness and positivity of the solution are discussed in the next section. In Section 3, local and global stabilities of infection-free equilibrium *E*_0_ are discussed. The stability of recombinant absent equilibrium *E*_1_ is presented in Section 4. Numerical simulation is discussed in Section 5. Finally, we have given conclusion in Section 6.

## 2. Positivity and Well-Posedness of the Solution

This section discusses the positivity and well-posedness of system (3).

**Theorem 1 .**

All the solutions of system (3) are nonnegative provided the initial conditions are nonnegative and bounded.

**Proof**

Consider *B* = *C*([−*τ*, 0]; *R*^5^) to be the Banach space of continuous mapping. These are the mappings from [−*τ*, 0] to *R*^5^ equipped with the sup-norm. For system (4), consider the initial conditions (*x*(*ϕ*), *y*(*ϕ*), *z*(*ϕ*), *v*(*ϕ*), *w*(*ϕ*)) ∈ *X*, satisfying

(4) xϕ≥0,yϕ≥0,zϕ≥0,vϕ≥0,wϕ≥0,ϕ∈−τ,0.

There exists unique solution (*x*(*t*), *y*(*t*), *z*(*t*), *v*(*t*), *w*(*t*)) of system (3) under the given initial conditions (4). By using constant of variation formula, we get the following solution of system (3):

(5) xt=x0e−∫0td+βvζdζ+λ∫0tβe−aτxt−τvt−τe−∫ηtd+βvζdζdη,yt=y0e−∫0ta+αzζdζ+∫0tβe−aτxt−τvt−τe−∫ηta+αvζdζdη,zt=z0e−bt+∫0tαwtyte−∫ηt−bt−ζdζdη,vt=v0e−pt+∫0tke−pt−ηdη,wt=w0e−qt+∫0tczηe−qt−ηdη,

which show the positivity of the solution. For boundedness of the solution (*x*(*t*), *y*(*t*), *z*(*t*), *v*(*t*), *w*(*t*)), we consider

(6) Mt=ckxt+ckyt+ckzt+ac2vt+bk2wt.

The derivative of (6) yields

(7) dMtdt=ckλ−dxt−βe−aτxt−τvt−τ+γyt+ckβe−aτxt−τvt−τ−ayt−γzt+ac2kyt−pvt+bk2czt−qwt=ckλ−dckxt+a2ckyt+b2ckyt−αwtvt+ckawtyt−bzt+qbk2wt+pac2vt≤ckλe−aτ−ΩMt.

Here *Ω* = min⁡{*d*, *a*/2, *b*/2, *q*, *p*}. This means that *M*(*t*) is bounded, so *x*(*t*), *y*(*t*), *z*(*t*), *v*(*t*), and *w*(*t*) are bounded.

System (4) has the following three possible biologically meaningful equilibria [17, 18]: disease-free equilibrium *E*_0_(*x*_0_, *y*_0_, *z*_0_, *v*_0_, *w*_0_), recombinant absent equilibrium *E*_1_(*x*_1_, *y*_1_, *z*_1_, *v*_1_, *w*_1_), and recombinant present equilibrium *E*_2_(*x*_2_, *y*_2_, *z*_2_, *v*_2_, *w*_2_) are given by

(8) E0=λd,0,0,0,0,E1=a+γpβke−aτ,λkβe−aτ−dpa+γkaβe−aτ,0,λkβe−aτ−dpa+γpaβe−aτ,0,E2=αλc+γbqpαcdp+βkqbe−aτ,bqαc,qαcckλαβe−aτ−αcdpa+γ−abqkβe−aταcdp+bkqβe−aτ,kqbαcp,αckβλe−aτ−αcdpa+γ−abqkβe−aτααcdp+bkqβe−aτ.

Each equilibrium point can be interpreted as follows. *E*_0_ is an infection-free equilibrium corresponding to maximal levels of healthy CD4 T cells. The second equilibrium *E*_1_ corresponds to positive levels of healthy CD4 T cells, infected cells, and virus, but no recombinant virus. The third equilibrium *E*_2_ corresponds to positive levels of healthy CD4 T cells, infected cells, virus, and recombinant virus.

The basic reproduction number (see [19]) is obtained from the proposed model as follows:

(9) $$\begin{array}{c} R_{0}=\frac{k\beta \lambda e^{-a\tau }}{dp\left(a+\gamma \right)}. \end{array}$$

For *R*_0_ < 1, *E*_0_ is the only equilibrium which is biologically meaningful. If *R*_0_ > 1, there is another equilibrium point *E*_1_. But *E*_2_ exists if and only if *R*_2_ > 1, where

(10) R2αβλcke−aτ−αcdpa+γβbkqa+γe−aτ=a+γαcdpβbkqe−aτR0−1.

Suppose that *R*_1_ = 1 + *βbkqe*^−*aτ*^/*αcdp*, and *R*_2_ > 1 if and only if *R*_0_ > *R*_1_.

## 3. Stability of the Disease-Free Equilibrium *E*_0_

The dynamical behavior of system (4) at *E*_0_ is discussed in this section.

**Theorem 2 .**

For *R*_0_ < 1, the disease-free equilibrium *E*_0_ is locally asymptotically stable while, for *R*_0_ > 1, *E*_0_ becomes unstable and the recombinant absent equilibrium *E*_1_ occurs.

**Proof**

After liberalization around *E*_0_ system (4) becomes

(11) x˙t=−dxt−βe−aτλdvt−τ+γyt,y˙t=βe−aτλdvt−τ−a+γyt,z˙t=−bzt,v˙t=kyt−pvt,w˙t=czt−qwt.

The characteristic equation corresponding to the Jacobian matrix of the linearized system (11) is given by

(12) b+ρd+ρq+ρ·a+γ+ρp+ρ−λdβke−τρ+a,

where *ρ* stands for eigenvalue. The first factor of the above equation has three negative roots and the nature of the roots of the second factor is discussed in the following:

(13) $$\begin{array}{c} \left(\;a+\gamma +\rho \;\right)\left(\;p+\rho \;\right)=\frac{\lambda }{d}\beta ke^{-\tau \left(\rho +a\right)}. \end{array}$$

The modulus of the left hand side of (13) satisfies

(14) $$\begin{array}{c} \left|\;\left(\;a+\gamma +\rho \;\right)\left(\;p+\rho \;\right)\;\right|\geq \left(\;a+\gamma \;\right)p, \end{array}$$

provided that *ρ* has nonnegative real part. The modulus of the right hand side of (13) gives

(15) $$\begin{array}{c} \frac{\lambda }{d}\beta k\left|\;e^{-\tau \left(\rho +a\right)}\;\right|=\left|\;\left(\;a+\gamma \;\right)pR_{0}\;\right|<\left(\;a+\gamma \;\right)p. \end{array}$$

But this is contradiction. Thus, when *R*_0_ < 1, then all the eigenvalues have negative real part. Thus the infection-free state *E*_0_ is locally asymptotically stable. For *R*_0_ > 1, we have

(16) $$\begin{array}{c} g\left(\;\rho \;\right)=\left(\;a+\gamma +\rho \;\right)\left(\;p+\rho \;\right)-\frac{\lambda }{d}\beta ke^{-\tau \left(\rho +a\right)}. \end{array}$$

Now *g*(0) = (*a* + *γ*)*p*(1 − *R*_0_) < 0 and lim_*ρ*→*∞*_*g*(*ρ*) = +*∞*. There exists at least one positive root of *g*(*ρ*) = 0. Therefore, the infection-free equilibrium *E*_0_ is unstable if *R*_0_ > 1 (see [20]).

**Theorem 3 .**

The disease-free equilibrium *E*_0_ is globally asymptotically stable when *R*_0_ < 1,

**Proof**

Consider

(17) V0t=12xt−λd2+λdyt+λdzt+a+γλkdvt+bλcdwt+βλde−aτ∫t−τtxζvζdζ,

where *V*_0_ stands for Lyapunov function. The derivative of (17) and the use of system (4) yield

(18) V˙0t=xt−λdλ−dxt−βe−aτxt−τvt−τ+γyt+λdβe−aτxt−τvt−τ−ayt−γyt+αwtyt+λdwtyt−bzt+a+γλkdkyt−pvt+bλcdczt−qwt+βλde−aτ∫t−τtxζvζdζ.

After further simplification, the above equation becomes

(19) V˙0t=−xt−λd·xt−λd+βe−aτxt−τvt−τ−λd−xtγy−qbλcdwt−a+γpλdkkβλe−aτa+γdp−1vt=−xt−λd·xt−λd+βe−aτxt−τvt−τ−λd−xtγy−a+γpλdk1−R0vt−qbλcdwt.

Thus, when *R*_0_ < 1, then (19) implies that ${\dot{V}}_{0}(t)<0$ and equality holds if and only if *x*_0_ = *λ*/*d*,  *y*(*t*) = 0,  *z*(*t*) = 0,  *v*(*t*) = 0,  *w*(*t*) = 0. Thus, by using LaSalle's invariance principle (see [21]), we conclude that *E*_0_ is globally asymptotically stable when *R*_0_ < 1.

## 4. Stability of Recombinant Absent Equilibrium *E*_1_

This section is devoted to the analysis of *E*_1_.

**Theorem 4 .**

If 1 < *R*_0_ < *R*_1_, then the recombinant present equilibrium *E*_1_ is locally asymptotically stable while *E*_1_ becomes unstable for *R*_0_ > *R*_1_.

**Proof**

The linearized form of model (4) at *E*_1_(*x*_1_, *y*_1_, *z*_1_, *v*_1_, *w*_1_) becomes

(20) x˙t=−dxt−βe−aτx1vt−τ+v1xt−τ+γyt,y˙t=βe−aτx1vt−τ+v1xt−τ−a+γyt−αy1wt,z˙t=αy1wt−bzt,v˙t=kyt−pvt,w˙t=czt−qwt.

Let *Z*_1_(*ρ*) *Z*_2_(*ρ*) = 0 be the characteristic equation of the Jacobian matrix of system (20), where

(21) Z1ρ=ρ2+b+qρ+bq−cαλkβe−aτ−dpa+γakβe−aτ,

(22) Z2ρ=ρ3+a+γ+p+kβλa+γpe−aτρ2+kβλa+γpe−aτa+γ+p+a+γpρ+kβλe−aτ−a+γρ+dpe−ρτ.

Now *Z*_1_(*ρ*) can be simplified as

(23) $$\begin{array}{c} Z_{1}\left(\;\rho \;\right)=\rho^{2}+\left(\;b+q\;\right)\rho +bq\left(\;1-R_{2}\;\right), \end{array}$$

which indicates that *Z*_1_(*ρ*) = 0 has two roots with negative real part if and only if *R*_2_ < 1 (i.e., *R*_0_ < *R*_1_) or one positive and one negative root if *R*_2_ > 1 (i.e., *R*_0_ > *R*_1_). Therefore, if *R*_0_ > *R*_1_, then the single infection equilibrium *E*_2_ is unstable. After some simplification *Z*_2_(*ρ*) = 0 can be written as

(24) $$\begin{array}{c} \rho^{3}+a_{2}\left(\;\tau \;\right)\rho^{2}+a_{1}\left(\;\tau \;\right)\rho +a_{0}\left(\;\tau \;\right)-\left(\;c_{1}\rho +c_{2}\;\right)e^{-\rho \tau }=0, \end{array}$$

where

(25) a2τ=a+γ+p+kβλa+γpe−aτ,a1τ=kβλa+γpe−aτa+γ+p+a+γp,a0τ=kβλe−aτ,c1=a+γp,c2=a+γpd.

It is clear that *ρ* = 0 is not a root of (24) if *R*_0_ > 1. When *τ* = 0, (24) becomes

(26) $$\begin{array}{c} \rho^{3}+a_{2}\left(\;0\;\right)\rho^{2}+\left(\;a_{1}\left(\;0\;\right)-c_{1}\;\right)\rho +a_{0}\left(\;0\;\right)-c_{2}=0. \end{array}$$

Using Routh-Hurwitz criterion (see [22]), we can prove that

(27) a20=a+γ+p+kβλa+γp>0,a10−c1=kβλa+γpa+γ+p>0,a00−c2=a+γpdR0τ=0−1>0.

Similarly,

(28) a20a10−c1−a00−c2=k2β2λ2a+γ2p2a+γ+p+kβλa+γpa+γ+p2+a+γpd>0.

Thus, any root of (24) has negative real part when *τ* = 0. Now we consider the distribution of the roots when *τ* > 0. Let *ρ* = *iκ*  (*κ* > 0) be the pure imaginary root of (24). Then, we obtain

(29) −iκ3−a2τκ2+ia1τκ+a0τ−ic1κ+c2e−iκτ=0.

The modulus of the above equation gives the following result:

(30) Hsκ2κ6+a22τ−2a1τκ4+a12τ−2a0τa2τ−c12κ2+a02τ−c22=0.

Since

(31) a22τ−2a1τ=a+γ2+p2+d2R02>0,a12τ−2a0τa2τ−c12=d2a+γ2+p2R02>0,a02τ−c22=a+γ2p2d2R02−1>0,

we see that all the coefficients of the above equation are positive which implies that the function *H*_*s*_(*κ*^2^) is monotonically increasing for 0 ≤ *κ*^2^ < *∞* with *H*_*s*_(0) > 0. Therefore, (30) has no positive roots if *R*_0_ > 1. Equation (24) has all the roots with negative real part if *τ* > 0 and *R*_0_ > 1.

**Theorem 5 .**

For 1 < *R*_0_ < *R*_1_, the recombinant present equilibrium *E*_1_ is globally asymptotically stable.

**Proof**

Let us construct the Lyapunov functional

(32) V1t=x−x1ln⁡x+y−y1ln⁡y+z+a+γkv−v1ln⁡v+bcw+x1v1βe−aτ∫t−τtxθvθv1xθ+τ−ln⁡xθvθdθ.

The derivative of (32) yields

(33) V˙1t=1−x1xx˙+1−y1yy˙+z˙+a+γk1−v1vv˙+bcw˙+x1v1βe−aτxtvtxt+τv1−xt−τvt−τxtv1−ln⁡xtvt+ln⁡xt−τvt−τ=1−x1xλ−dxt−βe−aτxt−τvt−τ+γyt+1−y1y·βe−aτxt−τvt−τ−a+γyt−αwtyt+αwtyt−bzt+a+γk1−v1vkyt−pvt+bcczt−qwt+x1v1βe−aτxtvtxτ+tv1−xt−τvt−τxtv1+lnxt−τvt−τxtvt.

Model (1) at single infection equilibrium *E*(*x*_1_, *y*_1_, *z*_1_, *v*_1_, *w*_1_) becomes

(34) λ=dx1+βe−aτx1v1+γy1,βe−aτx1v1=a+γy1,ky1=pv1.

If *τ* is very large, that is, when the time delay in the contact of uninfected targeted cells and pathogen virus is large and the latent period is very large, then the rate of infection will be very small and contrarily if *τ* is very small, then the infection will spread more rapidly. Therefore, we suppose that delay is very large, and taking limit we get

(35) $$\begin{array}{c} \underset{\tau \to \infty }{lim}\left(\;x\left(\;t+\tau \;\right)\;\right)=x\left(\;t\;\right). \end{array}$$

Using the above identities and assumption (33) in (35), we get

(36) V˙1t=dx12−xx1−x1x+βe−aτx1v13−x1x−yv1y1v−y1xt−τvt−τyx1v1+ln⁡xt−τvt−τxv−x1x−1γy+αdpβkR0−R1wt.

The following inequalities hold [14]:

(37) 2−xx1−x1x≤0,3−x1x−yv1y1v−y1xt−τvt−τyx1v1+ln⁡xt−τvt−τxv≤0.

Therefore, by using the above inequalities, (36) implies that *dV*_1_/*dt* < 0 when *R*_0_ < *R*_1_, and the equality holds when *x* = *x*_1_,  *y* = *y*_1_,  *z* = 0,  *v* = *v*_1_,  *w* = 0. Therefore, by LaSalle's invariance principle [21], we conclude that *E*_1_ is globally asymptotically stable.

## 5. Numerical Simulation

In this section, we present the numerical simulations by using MATLAB to illustrate our theoretical results. The drugs therapy can control the HIV-1. Using drugs therapy, the infected cells revert to the uninfected cells. For numerical simulation, we consider the values of the parameters presented in Table 1 [11].

**Figure 1:**
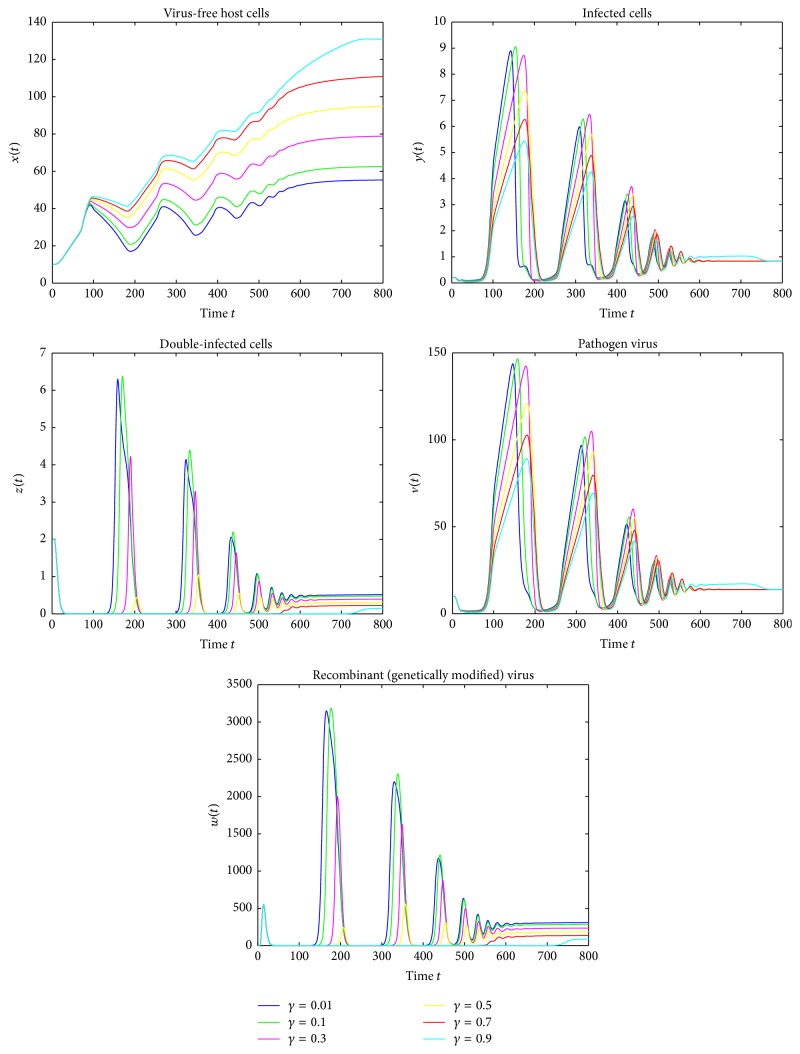
Simulation of system (4) for *τ* = 1.5, showing convergence to the stable equilibrium *E*_1_.

**Figure 2:**
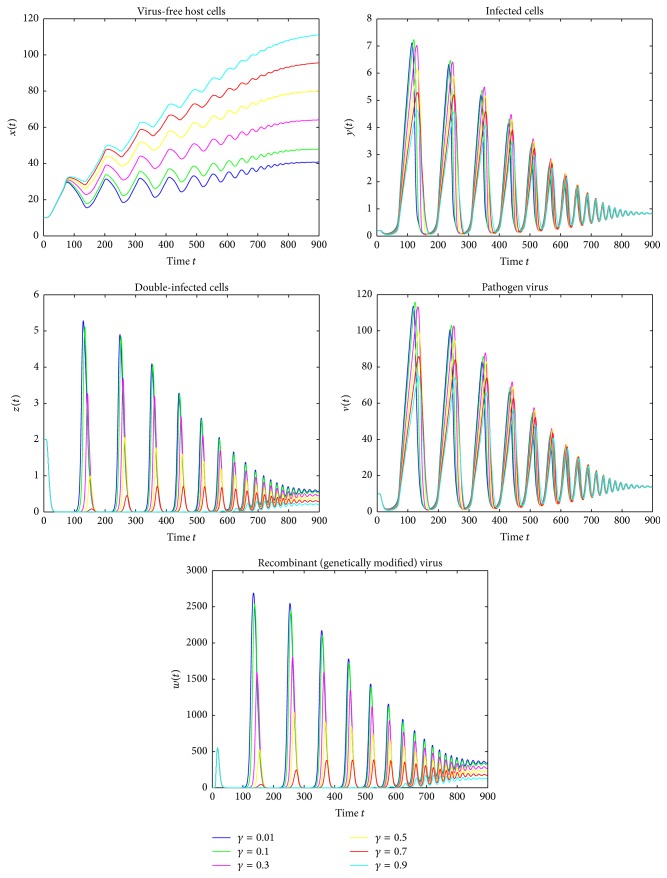
Simulation of system (4) for *τ* = 0.7 showing convergence to the stable equilibrium *E*_2_.

**Figure 3:**
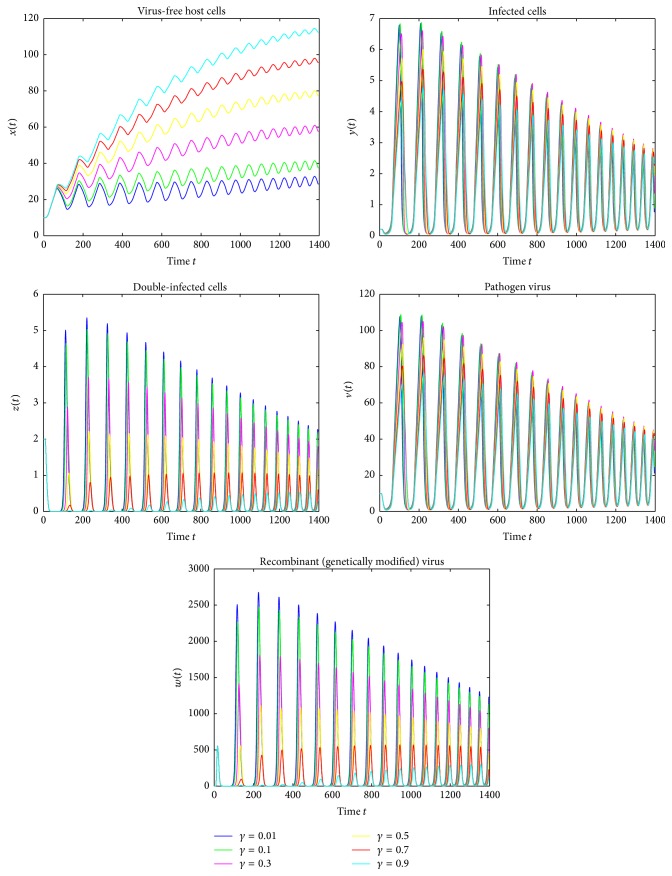
Simulation of system (4) for *τ* = 0.4 showing oscillating behavior.

Figures 1–3 are the oscillations of uninfected cells, infected cells, double-infected cells, pathogen virus, and recombinant virus.  Figure 1 shows the dynamical behavior of HIV-1 infection for the delay term *τ* = 1.5 and for different recovery rates *γ* = 0.01,0.1,0.3,0.5,0.7,0.9 and represents that as the value of the recovery rate increases the density of of uninfected cells increases and the concentration of infected cells decreases.  Figure 2 shows that by varying time delay *τ* = 0.7 and keeping the values of *γ* constant, the amplitude of oscillation increases and the rate towards stability decreases.  Figure 3 shows that if we further reduce the delay time *τ* = 0.4, then amplitudes of oscillations increases. More importantly, it is noted that the amplitudes of the oscillations in Figure 3 are almost double of that in Figure 1 though their frequencies are almost not changed. These figures show that introducing even very small time delay in the model can produce significant quantitative changes in solutions, which cannot be observed from the model without delay. Also, as the value of recovery rate increases the infected cells revert to the healthy cells more rapidly and converge to stable equilibrium. We can see that the infection would always keep stability when the cure rate *γ* is larger. Therefore, we can also claim that the cure rate *γ* is a very important parameter and by improving the cure rate, we will control the disease. Moreover, the significant qualitative changes due to existence of delay can be observed. These results also suggest that the delay is very important fact which should not be missed.

## 6. Conclusion

In this paper, a delayed HIV-1 model with drugs therapy is presented. The improved model with delay has three equilibrium solutions *E*_0_, *E*_1_, and *E*_2_. It has been shown that *E*_0_ is locally as well as globally asymptotically stable for *R*_0_ ∈ (0,1), which loses its stability at *R*_0_ = 1. Then, *E*_0_ bifurcates into *E*_1_. Next, it is also proved that *E*_1_ is also locally and globally asymptotically stable for *R*_0_ ∈ (1, *R*_1_). Delay, as the bifurcation parameter, plays a very important role in determining the dynamic behavior of the system. Delay may change the dynamical behavior quantitatively, even in the normal range of values. This indeed suggests that delay is a very important fact which should not be missed in HIV-1 modeling. The drugs therapy also has an important effect on model (3). As the value of recovery rate increased the infected cells revert to the uninfected cells resulting in decrease in infected cells and increase in healthy cells. And this infection can easily be controlled if we improve the cure rate.

## Figures and Tables

**Table 1:** Parameters values used for numerical simulation.

Parameters	Definition	Value (day^−1^)
*λ*	Generation rate of host cell	2 cells/mm^3^
*d*	Natural death rate of host cell	0.01
*β*	Rate of infection	0.004 mm^3^/vir
*a*	Death rate of HIV-1 infected cell	0.5
*α*	Rate of double infection	Assumed *α* = *β*
*b*	Death rate of double-infected cell	2
*k*	HIV-1 production rate by infected cells	50 vir/cell
*p*	Removal rate of HIV-1	3
*c*	Production rate of recombinant	2000 vir/cell
	by a double-infected cell	
*q*	Rate of removal of recombinant	Assumed *q* = *p*
*τ*	Delay	1.0~1.5 days

## References

[1] Perelson A. S., Kirschner D. E., Boer R. D. (1993). Dynamics of HIV infection of CD4^+^ T cells. *Mathematical Biosciences*.

[2] Perelson A. S., Neumann A. U., Markowitz M., Leonard J. M., Ho D. D. (1996). HIV-1 dynamics in vivo: virion clearance rate, infected cell life-span, and viral generation time. *Science*.

[3] Perelson A. S., Nelson P. W. (1999). Mathematical analysis of HIV-1 dynamics in vivo. *Society for Industrial and Applied Mathematics*.

[4] Rong L., Gilchrist M. A., Feng Z., Perelson A. S. (2007). Modeling within-host HIV-1 dynamics and the evolution of drug resistance: trade-offs between viral enzyme function and drug susceptibility. *Journal of Theoretical Biology*.

[5] Buonomo B., Vargas-de-León C. (2012). Global stability for an HIV-1 infection model including an eclipse stage of infected cells. *Journal of Mathematical Analysis and Applications*.

[6] Tian Y., Liu X. (2014). Global dynamics of a virus dynamical model with general incidence rate and cure rate. *Nonlinear Analysis. Real World Applications. An International Multidisciplinary Journal*.

[7] Zack J. A., Haislip A. M., Krogstad P., Chen I. S. Y. (1992). Incompletely reverse-transcribed human immunodeficiency virus type 1 genomes in quiescent cells can function as intermediates in the retroviral life cycle. *Journal of Virology*.

[8] Zack J. A., Arrigo S. J., Weitsman S. R., Go A. S., Haislip A., Chen I. S. Y. (1990). HIV-1 entry into quiescent primary lymphocytes: Molecular analysis reveals a labile, latent viral structure. *Cell*.

[9] Schnell M. J., Johnson J. E., Buonocore L., Rose J. K. (1997). Construction of a novel virus that targets HIV-1-infected cells and controls HIV-1 infection. *Cell*.

[10] Nolan C. P. (1997). Harnessing viral devices as pharmaceuticals: Fighting HIV-1's fire with fire. *Cell*.

[11] Revilla T., García-Ramos G. (2003). Fighting a virus with a virus: a dynamical model for HIV-1 therapy. *Mathematical Biosciences*.

[12] Jiang X., Yu P., Yuan Z., Zou X. (2009). Dynamics of an HIV-1 therapy model of fighting a virus with another virus. *Journal of Biological Dynamics*.

[13] Yu P., Zou X. (2012). Bifurcation analysis on an HIV-1 model with constant injection of recombinant. *International Journal of Bifurcation and Chaos*.

[14] Tian Y., Bai Y., Yu P. (2014). Impact of delay on HIV-1 dynamics of fighting a virus with another virus. *Mathematical Biosciences and Engineering*.

[15] Hattaf K., Yousfi N. (2012). Two optimal treatments of HIV infection model. *World Journal of Modelling and Simulation*.

[16] El Boukari B., Noura Y. (2014). A delay differential equation model of HIV infection, with therapy and CTL response. *Bulletin of Mathematical Sciences and Applications*.

[17] Ali N., Zaman G., Algahtani O. (2016). Stability analysis of HIV-1 model with multiple delays. *Advances in Difference Equations*.

[18] Culshaw R. V., Ruan S., Webb G. (2003). A mathematical model of cell-to-cell spread of HIV-1 that includes a time delay. *Journal of Mathematical Biology*.

[19] Herz A. V. M., Bonhoeffer S., Anderson R. M., May R. M., Nowak M. A. (1996). Viral dynamics in vivo: limitations on estimates of intracellular delay and virus decay. *Proceedings of the National Academy of Sciences of the United States of America*.

[20] Mittler J. E., Markowitz M., Ho D. D., Perelson A. S. (1999). Improved estimates for HIV-1 clearance rate and intracellular delay. *AIDS*.

[21] LaSalle J. P. (1976). *The Stability of Dynamical Systems*.

[22] Gantmacher F. R. (1960). *The Theory of Matrices*.

